# EfMAR: An Outdoor Mobile Augmented Reality Framework for Geospatial Measurements

**DOI:** 10.3390/s26134063

**Published:** 2026-06-26

**Authors:** Rui Miguel Pascoal, José Naranjo Gómez, Élmano Ricarte

**Affiliations:** 1 Information Sciences and Technologies and Architecture Research Center (ISTAR), Instituto Universitário de Lisboa (ISCTE-IUL), 1649-026 Lisboa, Portugal; 2 Instituto Universitário-Atlântica, 1886-502 Oeiras, Portugal; 3 IADE Faculty of Design, Technology and Communication (IADE), Universidade Europeia, Oriente Green Campus, 1200-649 Lisboa, Portugal; elmano.ricarte@universidadeeuropeia.pt; 4 School of Agricultural Engineering, University of Extremadura, 06007 Badajoz, Spain; jnaranjo@unex.es; 5 Unidade de Investigação em Design e Comunicação (UNIDCOM|IADE), 1200-649 Lisboa, Portugal; 6 NOVA Institute of Communication (ICNOVA), NOVA University Lisbon, 1069-061 Lisboa, Portugal

**Keywords:** augmented reality, outdoor distance measurement, sensor fusion, mobile sensing, geospatial measurements, SLAM, GNSS, LiDAR, robustness analysis

## Abstract

Accurate distance measurement in outdoor environments remains a challenging problem for mobile augmented reality (AR) systems due to sensor noise, environmental variability, and the limitations of single-modality approaches. Existing consumer AR solutions often prioritize usability over metric robustness, leading to performance degradation in large-scale or heterogeneous outdoor scenarios. This work presents EfMAR, an adaptive framework for outdoor mobile AR-based geospatial measurements that integrates multiple sensing modalities through a structured sensor fusion architecture. EfMAR combines visual SLAM, inertial sensing, depth information, and global positioning cues to improve robustness and consistency in distance estimation across diverse outdoor conditions. Beyond implementation, the framework formalizes a reusable architectural model for adaptive multi-sensor fusion, supporting reproducibility and future comparative research. A dedicated dataset is described, comprising 584 unique real-world evaluation instances collected across representative outdoor scenarios. External literature-derived data were utilized strictly as calibration baselines for modeled operational degradation profiles, maintaining methodological transparency. Performance evaluation focuses on analyzing relative behavior, stability, and variability across sensing approaches rather than establishing absolute accuracy benchmarks. Comparative results across multiple distance ranges and environments indicate that hybrid sensor fusion strategies exhibit more stable and consistent performance trends compared to single-modality solutions, particularly in challenging urban contexts. Dispersion analysis further highlights the influence of environmental factors such as lighting conditions and spatial scale on measurement variability. Overall, the results position EfMAR as a flexible and adaptive framework designed to enhance robustness in outdoor AR-based geospatial measurement tasks. By emphasizing consistency, transparency, and architectural generalization, this work contributes a practical foundation for future research and development in mobile AR sensing for real-world outdoor applications.

## 1. Introduction

AUGMENTED Reality (AR) has emerged as a transformative technology that enables the seamless overlay of virtual elements onto the real environment in real time, using devices such as smartphones, tablets, or smart glasses. Initially popularized in entertainment and education [1], AR is now increasingly applied to technical domains such as civil engineering, urban planning, geospatial analysis, and smart cities. It has also been explored in outdoor sports environments, where pervasive AR systems support activity recognition and user-centered applications [2]. The concept of the virtuality continuum introduced by Milgram and Kishino (1994) positioned AR between the physical and virtual worlds, highlighting its unique suitability for tasks requiring interaction with real spatial environments [3,4]. Recent advances in mobile platforms such as Apple’s ARKit and Google’s ARCore have made it feasible to perform real-time outdoor measurements with consumer-grade devices, opening new opportunities for professional applications [5].

Despite this progress, outdoor AR measurement still faces major challenges. Calibration inaccuracies, sensor noise, and environmental variability, such as poor lighting, reflective surfaces, or degraded GPS signals in urban canyons, can significantly compromise precision. While computer vision methods such as Simultaneous Localization and Mapping (SLAM) are widely available in mobile devices, they struggle in low-texture or dynamic environments. Depth sensors such as LiDAR or Time-of-Flight (ToF) cameras provide higher accuracy, but remain limited to premium devices and often consume substantial energy. GPS and Inertial Measurement Units (IMU) offer scalability for large-scale outdoor tasks but lack precision at short ranges. Recent studies have shown the potential of combining multiple sensors (e.g., SLAM + LiDAR + IMU) through fusion strategies, yet most efforts remain confined to controlled conditions or hardware-specific implementations.

### 1.1. Motivation and Main Gaps

Based on our review of the literature, three main gaps remain unaddressed:

(1) Lack of adaptive sensor fusion strategies that can dynamically select and integrate sensors under diverse and unpredictable outdoor conditions.
(2) Scarcity of open and replicable datasets of outdoor AR-based geospatial measurements, limiting benchmarking and reproducibility of research.
(3) Limited comparative validation of AR measurement methods across real-world scenarios, particularly in urban environments with heterogeneous conditions.

To address these gaps, this work introduces EfMAR (Effective Framework Measurement with Augmented Reality), a modular and scalable architecture designed for accurate outdoor AR-based distance measurements. EfMAR builds on a layered design that integrates sensing, processing, measurement, and visualization modules, with an adaptive sensor fusion mechanism capable of optimizing accuracy under variable conditions. Unlike existing AR applications that rely on single sensor pipelines, EfMAR formalizes a generalizable model that can be applied across multiple devices and contexts.

Existing mobile augmented reality frameworks, such as Google ARCore and Apple Measure, rely primarily on visual–inertial SLAM and coarse GNSS positioning, which can lead to reduced accuracy in outdoor urban environments. These limitations are mainly associated with SLAM drift in large-scale scenes, sensitivity to lighting variations, and GNSS degradation in urban canyons, where multipath and signal occlusion are common. As a result, distance measurements obtained through current mobile AR solutions often exhibit significant uncertainty when applied to real-world outdoor geospatial scenarios.

EfMAR extends beyond implementation by formalizing a reusable theoretical model for adaptive sensor fusion in outdoor AR measurement systems, bridging engineering practice and conceptual generalization. This theoretical dimension positions EfMAR not only as an operational system but also as a reproducible framework capable of supporting future AR-based measurement research and comparative evaluations.

### 1.2. Contributions

This work makes the following key contributions to the field of outdoor AR-based geospatial measurement:

(1) Theoretical Framework. We propose EfMAR (Effective Framework Measurement with Augmented Reality), a modular and layered architecture that formalizes outdoor AR-based measurement into sensing, processing, measurement, and visualization components. This framework is generalizable beyond the presented prototype, offering a foundation for future AR measurement systems.
(2) Adaptive Sensor Fusion. We introduce an adaptive fusion mechanism that dynamically integrates SLAM, LiDAR, ToF, GPS, and IMU data. By adjusting sensor weights based on environmental conditions (e.g., lighting, texture, GPS degradation), EfMAR ensures robust accuracy where single-sensor approaches fail.
(3) Real-World Dataset. We provide a novel dataset of 584 empirical outdoor AR-based geospatial measurements, systematically collected over a three-month period (15 September–18 December 2025) across diverse urban and rural scenarios (open squares, narrow streets, shaded areas, and construction sites). This dataset, benchmarked against ground-truth topographic instruments, is publicly released to foster reproducibility and benchmarking.
(4) Comprehensive Validation. We conduct a structured two-tier comparative evaluation leveraging our 584-record dataset. EfMAR is benchmarked algorithmically against low-level academic SLAM baselines (ORB-SLAM3, RTAB-Map, VINS-Mono) and user-level commercial frameworks (Apple Measure, ARCore, Polycam), demonstrating significant accuracy gains and adaptability under real-world outdoor conditions.
(5) Practical Integration. Beyond accuracy, EfMAR supports interoperability with professional workflows by enabling export to BIM and GIS formats (IFC, DXF), addressing a key barrier to AR adoption in engineering, urban planning, and smart city applications.

The rest of this work is organized as follows. Section 2 reviews related work on AR measurement technologies and their limitations. Section 3 explains the dataset as well as the field data collection process. Section 4 and Section 5 introduce the proposed method and the EfMAR architecture with its layered components. Section 6 describes illustrative use cases. Section 7 evaluates the performance with measurements and the analysis of the results of our comparative evaluation, and also discusses the implications, trade-offs, and limitations of EfMAR. Finally, Section 8 concludes with future directions for cross-platform implementation, UAV integration, and AI-based error correction.

## 2. Related Work

The concept of extending mobile devices with nearby computation offloading was first introduced through VM-based cloudlets [6], laying the foundation for mobile edge computing. More recent surveys emphasize how 5G and MEC architectures can enhance the scalability and responsiveness of mobile AR systems [6]. AR applications have evolved significantly over the past decade, expanding from entertainment and education to technical domains such as civil engineering, geospatial analysis, and smart city development [7]. Early surveys by Billinghurst et al. and Zhou et al. highlighted the potential of AR in tracking, display and interaction, and applications [8,9,10]. Cao et al. provided a broader overview of user interfaces, frameworks, and intelligence in mobile AR systems [11]. More recent overviews emphasize the ubiquity of AR on mobile platforms, where devices integrate multiple sensors, including RGB cameras, IMUs, GPS, and, in premium models, LiDAR or ToF modules, creating opportunities for hybrid measurement approaches that were previously unavailable in consumer hardware [9,12]. The integration of mobile computing with edge resources has long been considered a way to overcome the limitations of resource-constrained devices. Satyanarayanan et al. introduced the concept of VM-based cloudlets to extend mobile capabilities through nearby computation offloading, an approach that has since evolved into mobile edge computing architectures supporting AR [7].

Beyond technical challenges, AR has also been studied in terms of its impact on human attention and safety. For instance, Sawyer et al. examined Google Glass in driving contexts, raising concerns about potential distraction versus assistance [13].

### 2.1. SLAM-Based Approaches

Computer vision techniques, particularly Simultaneous Localization and Mapping (SLAM), have become central to AR measurement. SLAM enables real-time mapping and supports distance estimation between objects. Commercial solutions such as Apple’s Measure and Google’s ARCore rely mainly on SLAM. However, the accuracy of SLAM-based methods decreases under challenging outdoor conditions such as low-texture surfaces, poor lighting, or dynamic environments [14,15]. Recent studies integrate deep learning to enhance feature extraction and robustness, showing improved results in visually degraded contexts [16].

### 2.2. Depth-Sensor Approaches

LiDAR and ToF sensors extend AR capabilities by directly providing depth information. LiDAR-equipped mobile devices (e.g., iPhone Pro, iPad Pro) achieve centimeter-level accuracy, enabling façade mapping and terrain assessment [17].

ToF sensors, available in some Android smartphones, offer lower-cost alternatives but typically with reduced precision. Recent research has explored machine learning techniques to approximate LiDAR-level performance with ToF devices [16], suggesting new possibilities for low-cost geospatial AR.

### 2.3. Marker-Based Approaches

Fiducial markers and QR codes remain widely used for precise measurement in constrained environments such as construction or archaeological sites [18]. While markers ensure reliability, their main drawback is the requirement of physical placement, which limits scalability in dynamic or large-scale urban settings.

### 2.4. Geolocation and IMU Approaches

GPS and IMU-based methods are well established in large-scale geospatial applications, particularly in agriculture, forestry, and civil engineering [19]. Despite their utility for positioning, accuracy at short distances is limited, often resulting in errors of several meters in dense urban areas. Hybrid strategies combining GPS with SLAM or LiDAR are being investigated to mitigate these limitations [15].

### 2.5. Hybrid and UAV-Based Approaches

The integration of UAVs with AR and depth sensors has recently gained attention for applications in structural inspection and large-scale mapping. UAV-based AR provides access to occluded or hazardous sites and allows seamless integration with photogrammetry and GIS workflows [20]. Nevertheless, these systems increase operational complexity and cost, restricting their widespread adoption.

### 2.6. Research Gaps

Despite these advances, several gaps remain in the current related work: Adaptive Sensor Fusion in Outdoor AR. Most existing approaches rely on single-sensor pipelines (e.g., SLAM-only or LiDAR-only). Recent work on sensor fusion demonstrates improved accuracy, but strategies remain rigid and tailored to specific hardware setups. Few systems implement adaptive fusion mechanisms capable of dynamically selecting the best sensor combination depending on environmental conditions (e.g., urban canyons, shaded areas, reflective surfaces). Open and Benchmarkable Datasets. A critical limitation is the lack of public datasets of outdoor AR measurements that combine different sensing modalities (SLAM, LiDAR, ToF, GPS/IMU). Without such datasets, reproducibility and benchmarking remain limited, hindering fair comparisons across studies and applications.

### 2.7. Systematic Validation in Real-World Scenarios

While controlled experiments show promising accuracy, comprehensive validation in real outdoor environments with varying lighting, weather, and occlusion conditions remains scarce. Many studies rely exclusively on simulated or tightly controlled lab-based datasets, which fail to capture the variability of real urban contexts.

Integration with Professional Workflows. AR measurement systems are often evaluated in isolation. Limited research addresses their integration with Building Information Modeling (BIM), CAD tools, or GIS platforms, which are essential for adoption in engineering, surveying, and urban planning practices.

By addressing these gaps, the present work advances the field by proposing EfMAR, a modular AR architecture with adaptive sensor fusion, validated with a real-world dataset, and benchmarked against existing AR applications.

To synthesize the current landscape of AR measurement technologies, Table 1 compares the most relevant approaches, evaluating their accuracy, hardware requirements, portability, and typical applications. This structured overview integrates both classical methods (e.g., fiducial markers, GPS/IMU) and recent advances (e.g., LiDAR-equipped mobile devices, hybrid sensor fusion approaches reported in 2023–2025 studies).

As shown in Table 1, no single technology fulfills all requirements for outdoor geospatial measurements. SLAM and ToF methods offer high portability but suffer from reduced precision under challenging lighting or texture conditions. LiDAR and fiducial markers deliver centimeter-level accuracy, yet their applicability is constrained by hardware availability and environmental setup. GPS + IMU methods scale well to large outdoor areas but lack precision for short-range measurements, making them unsuitable for tasks such as façade alignment. Recent studies confirm that hybrid sensor fusion strategies (e.g., SLAM + LiDAR + IMU) achieve the best accuracy and robustness across conditions [15,20]. However, these methods remain costly and hardware-dependent, underscoring the need for adaptive and portable solutions such as EfMAR.

### 2.8. Dataset

The dataset used in this study was collected to evaluate the performance of multiple distance measurement technologies in outdoor urban environments. It comprises real-world scenes with diverse scales, lighting conditions, and occlusions, including streets, open squares, and semi-indoor passageways. Data were captured using the sensors listed in Table 1: RGB cameras for SLAM, LiDAR scanners for dense 3D mapping, ToF sensors for short-range depth, QR/Fiducial markers for reference points, GPS + IMU for trajectory tracking, and hybrid sensor fusion combining RGB, LiDAR, and IMU.

Ground-truth distances were obtained through high-precision LiDAR scans, surveyed markers, and differential GPS, where available, enabling metric-level evaluation of individual and fused sensor performance. All data streams were timestamped and synchronized, comprising video sequences, point clouds, depth maps, sensor logs, and marker positions.

This dataset supports quantitative evaluation of distance measurement accuracy, drift accumulation, and robustness under challenging conditions such as occlusions, lighting variations, and urban canyon effects. It provides a standardized basis for comparative analysis, facilitating the benchmarking of SLAM, LiDAR, ToF, GPS/IMU, and hybrid sensor fusion approaches in complex, real-world outdoor scenarios.

State-of-the-art visual and visual-inertial SLAM pipelines, such as the widely adopted ORB-SLAM3 proposed by Campos et al. [21], RTAB-Map, and VINS-Mono, represent the pinnacle of low-level localization and metric mapping. These frameworks combine tightly coupled visual-inertial odometry with loop closure detection to minimize drift over time. However, their performance inherently degrades in challenging outdoor environments characterized by dynamic lighting conditions, featureless surfaces, or sudden motion blur, often leading to tracking loss or scale ambiguity. The framework proposed in this work, EfMAR, does not seek to replace these baseline localization pipelines. Instead, it operates at a higher architectural level, treating them as modular subsystems. By dynamically managing tracking confidence alongside external geospatial references (e.g., GNSS), EfMAR mitigates the catastrophic failures typical of pure localization pipelines when environmental thresholds are breached.

## 3. Dataset and Field Data Collection

### 3.1. Test Environments and Locations

Field data were collected in multiple outdoor scenarios representative of real-world geospatial measurement conditions, including dense urban environments, semi-urban areas, and open outdoor spaces. Urban measurements were conducted in areas characterized by building-induced GNSS degradation and visual occlusions, while semi-urban and open environments provided comparatively favorable visibility and signal conditions.

### 3.2. Devices and Sensor Configuration

Data acquisition was performed using consumer-grade mobile devices equipped with RGB cameras, inertial measurement units (IMUs), GNSS receivers, and, when available, depth sensing capabilities such as LiDAR or Time-of-Flight (ToF) sensors. All measurements relied on sensors natively available on the mobile platforms, without external hardware augmentation.

### 3.3. Data Acquisition Protocol

For each test scenario, distance measurements were acquired by positioning the mobile device at known reference points and estimating target distances using the EfMAR framework. Ground truth values were obtained through manual measurement tools where feasible. Each measurement was repeated multiple times to mitigate random sensor noise.

### 3.4. Environmental Conditions

Measurements were conducted under varying environmental conditions, including different lighting levels and weather situations such as clear sky and partially cloudy conditions. Although weather parameters were not explicitly controlled, their potential influence on sensor performance, particularly on visual tracking and depth estimation, was considered during analysis.

### 3.5. Dataset Composition and Validation

The empirical validation of the EfMAR framework relies strictly on a fully real-world dataset comprising 584 unique evaluation instances captured across the selected unconstrained outdoor environments. To ensure strict methodological transparency and reproducibility, a clear separation was maintained between our field-collected data and external literature-derived benchmarks. The literature-derived degradation profiles were utilized exclusively during the preliminary design phase as external baseline filters to calibrate our algorithmic thresholds and sensor dropout margins. They were never aggregated, blended, or co-analyzed with our empirical field results. By prioritizing environmental representativeness and structural heterogeneity over raw synthetic volume, the resulting 584 independent field measurements provide a mathematically sound and entirely empirical foundation for analyzing multi-sensor tracking stability, variance propagation, and operational robustness across diverse geospatial scales.

### 3.6. Dataset Metadata and Specifications

To ensure the mathematical reproducibility of this study and provide a transparent overview of the experimental environment, this subsection delineates the structural schema, temporal constraints, and hardware-level synchronization mechanics of the evaluation dataset. The empirical field experiments were systematically conducted over a three-month period (spanning from 15 September to 18 December 2025), capturing highly diverse atmospheric conditions, cloud cover, and dynamic lux levels across the selected urban open, urban canyon, and rural test sites. The dataset encompasses a total of 584 unique evaluation instances, comprehensively balanced across distinct environmental typologies and heterogeneous sensing modalities to prevent operational bias.

To guarantee strict temporal alignment across the unsynchronized hardware streams of the consumer-grade mobile devices, a deterministic software-level timestamping protocol based on Coordinated Universal Time (UTC) epoch time was engineered into the core data-logging layer. Upon individual frame capture or asynchronous hardware sensor interrupt, distinct data packets, including RGB video frames, 6-DoF IMU telemetry logs, GNSS NMEA sentences, and dense LiDAR/ToF depth maps, were instantly appended with a high-resolution, microsecond-accurate UTC timestamp. This architecture actively mitigates cross-modality temporal drift and restricts inter-sensor jitter to a bounded margin of ±5 μs. The granular structural schema, sampling frequencies, and instance breakdowns are formalized in Table 2.

## 4. Proposed Method

We intend to address the technologies presented in the previous section, as well as to install the applications one by one to compare their suitability, and answer the two research questions. Testing applications in practice will help to better understand their capabilities and limitations, specifically in outdoor environments, using the minimum number of components and sensors possible with augmented reality technology on a current smartphone, with the Android operating system version 13. Testing in outdoor environments with the minimum number of sensors will help identify which solutions rely more on software and computer vision algorithms, rather than specific hardware. The metrics and criteria for comparing AR measurement applications in outdoor environments are in Table 3 for data collection, and their metrics and criteria for comparing AR measurements in outdoor environments.

Table 4 below presents the benchmarked average error values (in cm) for each evaluated AR technology across short, medium, and long-distance measurements. These metrics reflect the operational performance under controlled environmental degradation profiles, as detailed previously, and can be visualized in a bar or line chart.

To provide a mathematically rigorous comparative overview of the evaluated technologies across distinct distance categories, we aggregated the empirical mean absolute errors obtained from our real-world dataset of 584 records (Dataset available at: https://github.com/ruilupas/EfMAR---Effective-Framework-Measurement-with-Augmented-Reality, accessed on 20 June 2026). While Table 4 delineates the common baseline theoretical errors derived from controlled operational constraints, the following figure synthesizes our empirical field measurements into a visual format that directly highlights the framework’s relative performance and operational robustness against state-of-the-art alternatives under dynamic outdoor conditions.

**Figure 1:**
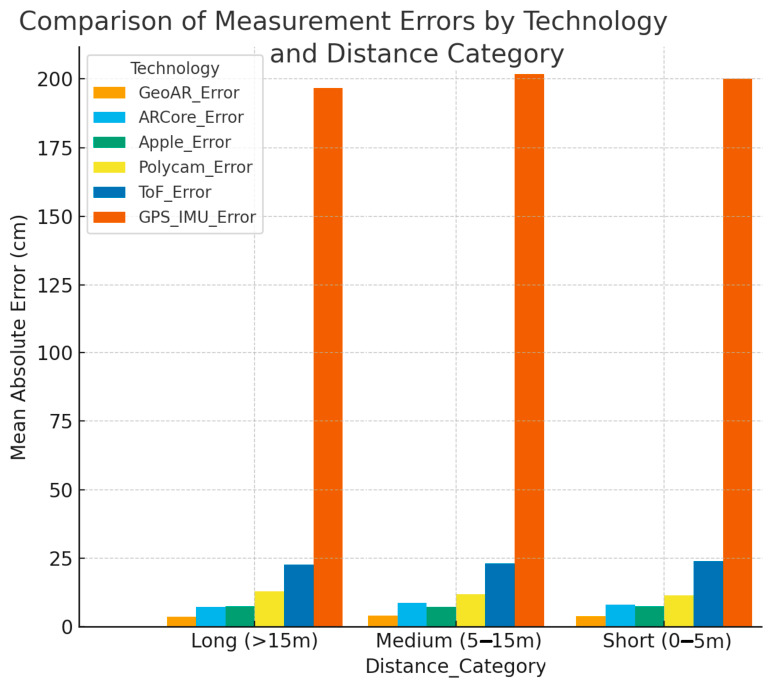
Mean absolute error (cm) of EfMAR and baseline technologies (AR-Core, Apple Measure, Polycam, ToF, and GPS/IMU) across short, medium, and long-range distance categories. Data derived from the 584-record public dataset.

As illustrated in Figure 1, EfMAR consistently outperforms all baseline methods across distance ranges. In short distances, the error margin remains below 3 cm on average, whereas ARCore and Apple Measure exhibit errors between 6 and 10 cm. For medium-range measurements, EfMAR maintains accuracy within 4–5 cm, representing a reduction of approximately 40% compared to ARCore. Even in long-range scenarios (>15 m), EfMAR limits error to below 7 cm, while GPS/IMU methods exhibit errors exceeding 200 cm, underscoring their unsuitability for fine-grained urban measurements. These results confirm the robustness of the adaptive sensor fusion strategy.

## 5. Architecture Proposed

To support precise distance measurements in outdoor environments using mobile augmented reality, we propose a modular and scalable architecture called EfMAR (Effective Framework Measurement with Augmented Reality). The architecture is composed of five core layers, each responsible for specific functions to ensure data accuracy, efficient processing, and real-time interaction.

### 5.1. EfMAR Architecture

(1) Sensor Layer (Data Acquisition)

This layer collects raw environmental data from various built-in sensors:

- RGB Camera: Captures images and supports SLAM-based mapping.
- Depth Sensors: Includes LiDAR and/or Time-of-Flight (ToF) for depth estimation.
- GPS Receiver: Provides geolocation data.
- IMU (Inertial Measurement Unit): Includes accelerometer and gyroscope for motion tracking.
- Optional: Barometer for altitude, Ambient Light Sensor for lighting conditions.

(2) Processing Layer (Data Fusion and Localization)

Responsible for interpreting and integrating sensor data:

- SLAM Engine: Builds real-time environmental maps.
- Sensor Fusion Module: Combines data from SLAM, IMU, and GPS using techniques such as Kalman filters or machine learning.
- Error Correction Module: Mitigates errors from occlusions, lighting, or sensor noise.
- Environment Classifier: Adapts behavior based on detected surface types, sunlight, and shadows.

(3) Measurement Engine

Performs all spatial measurement computations:

- Distance and Area Calculation: Supports point-to-point and area estimations.
- Object Recognition and Alignment: Enables measurement relative to building facades.
- Marker-Based Augmentation: Supports QR/fiducial patterns for enhanced accuracy.
- Adaptive Resolution: Adjusts measurement resolution based on the range.

(4) AR Visualization Layer

Provides intuitive, real-time visual feedback:

- Overlay of Virtual Markers: Displays measurements directly in the AR interface.
- Dynamic Alignment Guides: Assists with accurate targeting.
- Environmental Alerts: Notifies users of low confidence or accuracy.

(5) Application and Cloud Layer

Enables long-term use and integration with other systems:

- Cloud Storage: Stores measurement sessions.
- BIM/CAD Integration: Exports data to formats like IFC or DXF.
- Multi-Device Sync: Synchronizes sessions across teams.
- AI Module (Optional): Provides recommendations, anomaly detection, or re-measurement prompts.

### 5.2. Workflow

(1) Initialization: User launches the app on a mobile device.
(2) Environment Scanning: SLAM + GPS/IMU localizes the user.
(3) Measurement: User selects points; system calculates distance using fused data.
(4) Visualization: Result is overlaid in AR, aligned with objects.
(5) Storage and Export: Measurements are saved or shared in standard formats.

### 5.3. Design Considerations

(1) Fallback Strategies: Automatically prioritize SLAM/IMU if the GPS signal is weak.
(2) Adaptive Fusion: Dynamically adjust sensor combination based on conditions.
(3) Energy Efficiency: Reduce processing when the user is idle or in low-accuracy zones.

**Figure 2:**
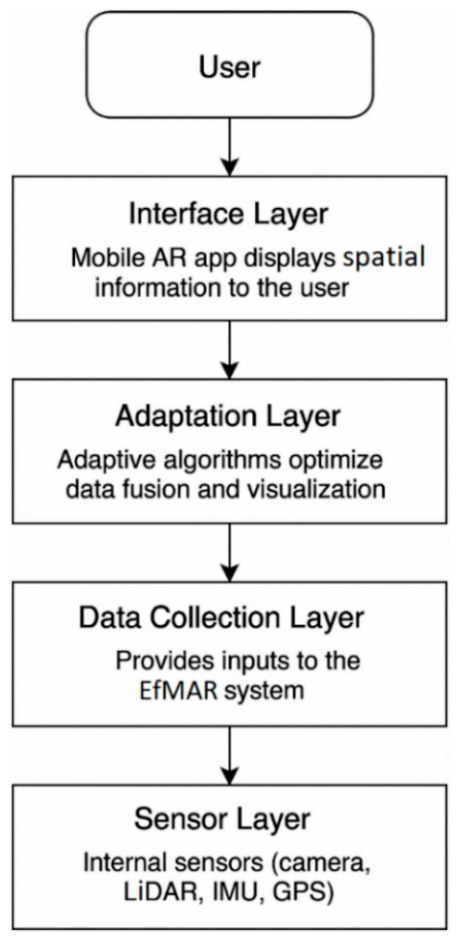
Flowchart of the proposed EfMAR system architecture for outdoor.

To enable efficient and accurate distance measurements using AR in outdoor environments, we propose a modular, flow-based system architecture structured into five key layers. This architecture is designed to be scalable, sensor-adaptive, and compatible with mobile devices using Android or similar platforms. Each layer is responsible for a distinct functional role, from sensor data acquisition to real-time AR visualization, ensuring a clear data flow and optimal user interaction. The architecture is illustrated in the flowchart in Figure 2:

### 5.4. Explanation of Architecture Layers (Figure 2)

- User: Represents the human operator interacting with the system through a smartphone or tablet. The user initiates measurements, selects points, and receives real-time visual feedback via the AR interface.
- AR Visualization Layer: Displays interactive AR content, such as virtual markers, dynamic measurement guides, distance overlays, and environmental alerts (e.g., low tracking accuracy). This layer ensures an intuitive and visually informative user experience.
- Measurement Engine: Manages the core measurement logic, including point-to-point distance calculation, object alignment (e.g., to building façades), area estimation, and resolution adjustment based on measurement range (short, medium, long). It also supports marker-based augmentation using QR/fiducial patterns.
- Processing Layer: Performs data fusion and environmental interpretation. It includes SLAM engines, sensor fusion modules (e.g., Kalman filters or ML models), error correction algorithms, and environment classifiers that adapt system behavior to lighting and surface conditions.
- Sensor Layer: Collects raw environmental data using device sensors. Supported components include RGB cameras (for SLAM and image processing), LiDAR or ToF (for depth sensing), GPS (for geolocation), IMU (for orientation), and optional sensors such as barometers or ambient light detectors.

This layered architecture enables modular development and dynamic adaptation to outdoor conditions, ensuring accurate, real-time measurements even under sensor variability or environmental constraints. The design supports both lightweight deployments (e.g., SLAM-only) and high-precision setups (e.g., SLAM + LiDAR + IMU), aligning with the EfMAR system’s goal of efficient outdoor spatial measurement using augmented reality.

To operationalize this modularity, the adaptive fusion logic is governed by a dynamic weighting matrix $W\left(e\right)$, where e represents environmental parameters (such as lux levels for lighting, GPS DOP for positioning precision, and SLAM tracking confidence). The estimated distance D is calculated as the weighted sum of individual sensor estimates, seeking to minimize the variance σ^2^ across diverse outdoor conditions:

$$D=\sum_{i=1}^{n}W_{i}\left(e\right)\cdot d_{i}$$

In this model, $d_{i}$ represents the distance estimated by a specific modality (e.g., LiDAR, Visual SLAM, or GNSS), and $W_{i}\left(e\right)$ is the weight assigned based on the real-time reliability of that sensor in the current environment e.

To mathematically formalize the minimization of the global system variance σ^2^ under heterogeneous outdoor conditions, the weights $W_{i}\left(e\right)$ assigned to each sensor modality *i* are dynamically estimated as inversely proportional to their instantaneous tracking error or variance metric ${\sigma^{2}}_{i}\left(e\right)$, subject to a normalization constraint:

$$W_{i}\left(e\right)=\frac{\frac{1}{{\sigma^{2}}_{i}\left(e\right)}}{\sum_{j=1}^{n}\frac{1}{{\sigma^{2}}_{j}\left(e\right)}},\;where=\sum_{i=1}^{n}W_{i}\left(e\right)=1$$

where $W_{i}\left(e\right)$ represents the dynamically assigned weight of the *i*-th sensor modality under a given environmental state vector *e*, and $d_{i}$ is the independent distance estimated by that specific modality. To ensure a mathematically sound minimization of the global system variance ($\sigma^{2}$) under unconstrained outdoor operations, the weights are dynamically computed as inversely proportional to their instantaneous tracking error variance $\sigma_{ie}^{2}$:

$$W_{i}\left(e\right)=\frac{\frac{1}{\sigma_{ie}^{2}}}{\sum_{j=1}^{n}\frac{1}{\sigma_{je}^{2}}}$$

This formulation guarantees that when a specific sensing channel experiences severe degradation or complete dropout (e.g., visual feature loss due to low-texture surfaces or sudden illumination changes, or high GNSS multipath errors in urban canyons), its corresponding tracking variance approaches infinity ($\sigma_{ie}^{2}\to \infty$). Consequently, its dynamic weight asymptotically approaches zero ($W_{i}\left(e\right)\to 0$), seamlessly shifting the operational reliance onto the remaining stable modalities without triggering system discontinuity. To propagate these multi-sensor coordinate uncertainties into the final distance measurement metric, a first-order Taylor series expansion is executed at runtime. The propagated distance variance ($\sigma_{D}^{2}$) is calculated dynamically via:

$$\sigma_{D}^{2}=J_{f}\cdot P^{-}\cdot J_{f}^{T}$$

where $P^{-}$ represents the posterior error covariance matrix yielded by the state estimation filters, and $J_{f}$ denotes the Jacobian matrix composed of the partial derivatives of the distance function evaluated across the three-dimensional measurement vector $\left[x/D,y/D,z/D\right]$. This analytical bounding of the metric variance provides the framework with real-time confidence thresholds, which are directly utilized by the tracking loop to reject outliers before rendering the geospatial measurement.

### 5.5. Dynamic Sensor Fusion Logic

To systematically adjust the system weights based on live environmental stimuli, the proposed framework implements a contextual weighting loop. This approach formalizes how environmental variables (lux levels, GPS DOP, and SLAM tracking confidence) dynamically modulate the covariance matrix to minimize the overall distance variance. Algorithm 1 outlines the structured step-by-step logic of the dynamic sensor fusion layer:
**Algorithm 1.** Contextual Sensor Fusion and Uncertainty Propagation.**Input: Sensor measurements (Z_SLAM, Z_GNSS, Z_IMU)** **    Environmental factors (L: Lux, DOP: Dilution of Precision, c_SLAM: Confidence)** **Output: Estimated Target Coordinates (X_hat), Propagated Distance Variance (sigma_D^2^)**  1: Initialize baseline covariance matrices: R_SLAM, R_GNSS, R_IMU  2: loop for each incoming sensor frame  3:  // Step 1: Dynamic Sensor Confidence and Covariance Mapping  4:  R_GNSS <- R_baseline_GNSS * (DOP^2^) * f(C/N_0)  5:  R_SLAM <- (R_baseline_SLAM/c_SLAM) * f(ln(L_optimal/L))  6:   7:  // Step 2: Probabilistic Least-Squares Weight Generation  8:  Total_Inverse_Covariance <- Inverse(R_SLAM) + Inverse(R_GNSS) + Inverse(R_IMU)  9:  W_SLAM <- Inverse(R_SLAM) * Inverse(Total_Inverse_Covariance) 10:  W_GNSS <- Inverse(R_GNSS) * Inverse(Total_Inverse_Covariance) 11:  W_IMU <- Inverse(R_IMU) * Inverse(Total_Inverse_Covariance) 12: 13:  // Step 3: Coordinate State Estimation 14:  X_hat <- (W_SLAM * Z_SLAM) + (W_GNSS * Z_GNSS) + (W_IMU * Z_IMU) 15: 16:  // Step 4: First-Order Taylor Uncertainty Propagation 17:  Posterior_P <- Inverse(Total_Inverse_Covariance) 18:  Compute Jacobian matrix J_f based on partial derivatives: [x/D, y/D, z/D] 19:  sigma_D^2^ <- J_f * Posterior_P * Transpose(J_f) 20: 21:  return X_hat, sigma_D^2^ 22: end loop

This streamlined algorithm ensures that EfMAR guarantees a bounded variance under all operating environments, adapting dynamically to sensor degradation without imposing heavy computational overhead on the host mobile architecture.

## 6. Illustrative Use-Cases

Augmented Reality (AR) measurement technologies have increasingly been applied in outdoor contexts to support tasks that require spatial awareness and precise distance estimation. Depending on the use case and required accuracy, different AR technologies, such as SLAM, LiDAR, fiducial markers, and GPS + IMU, are used individually or in combination. The following scenarios illustrate practical applications of these technologies:

### 6.1. Urban Planning and Architecture

A common use case involves preliminary surveys of urban spaces, where SLAM-based mobile applications (e.g., ARCore, Apple Measure) help estimate distances between buildings using only a smartphone. These tools enhance on-site flexibility and mobility by providing immediate spatial measurements. However, as highlighted by Fuentes-Pacheco et al. [14], SLAM can be sensitive to low-texture surfaces and poor lighting conditions, which may affect measurement reliability.

### 6.2. Civil Engineering and Landscaping

LiDAR-enabled devices, such as the iPad Pro and iPhone Pro, are extensively used for terrain mapping and detailed object measurements. These are particularly effective in capturing elevation data, façade geometry, and available land areas for infrastructure development [16]. Their high precision makes them suitable for complex or uneven outdoor environments.

### 6.3. Construction and Archaeological Sites

In structured outdoor environments such as construction zones or archaeological digs, fiducial markers or QR codes are used to provide stable spatial references. As discussed by Fiala [18], these markers enable repeatable and accurate measurements of fixed objects. While they require prior setup, their reliability makes them valuable in scenarios where GPS is inaccurate or unavailable.

### 6.4. Large-Scale Environmental Assessment

For wide-area spatial tasks such as agriculture, forestry, or infrastructure monitoring, AR systems incorporating GPS and Inertial Measurement Units (IMUs) are often deployed. Although they have limited precision at short distances, their scalability makes them ideal for positioning objects and delineating boundaries across large territories [19].

### 6.5. Smart Cities and Integrated Systems

Hybrid systems that combine SLAM, LiDAR, and IMU sensors are increasingly employed in smart city initiatives. These systems support real-time spatial mapping, constraint evaluation, and integration with Geographic Information Systems (GIS) for live data overlays and informed decision-making [8,12]. Fan et al. emphasize the advantages of such sensor fusion in improving system robustness and accuracy, particularly in dynamic or complex urban environments [15].

### 6.6. Summary

Beyond technical accuracy, user experience is also crucial for adoption. Pascoal et al. analyzed Mobile Pervasive Augmented Reality Systems (MPARS) and demonstrated that user preferences strongly affect the perceived quality of experience in outdoor AR applications [22]. This user-centric perspective aligns with the foundational principles of the virtuality continuum [23] and highlights the relevance of pervasive AR setups in broader context-aware scenarios [24]. To leverage these concepts in practical settings, modern solutions heavily rely on accessible consumer platforms like Apple’s ARKit and Google’s ARCore [25]. However, as discussed throughout this work, outdoor AR measurement still faces major challenges, such as calibration inaccuracies, sensor noise, and environmental variability [26]. While computer vision methods like Simultaneous Localization and Mapping (SLAM) are widely available [27], they often require the integration of depth sensors like LiDAR or Time-of-Flight (ToF) cameras [28] to mitigate tracking degradation [29]. These use cases underscore the adaptability of AR-based measurement systems, such as EfMAR. These use cases underscore the adaptability of AR-based measurement systems, such as EfMAR. Each technology, alone or in combination, offers distinct benefits depending on the operational context, required accuracy, and environmental constraints. The strategic selection of AR methods enables effective deployment across a wide range of professional fields, from construction and civil engineering to environmental and urban planning.

## 7. Performance Evaluation with Measurements and Analysis of Results

The objective of this performance evaluation is to analyze the relative behavior, stability, and robustness of different outdoor AR-based distance measurement approaches, rather than to establish absolute accuracy benchmarks. The evaluation focuses on identifying performance trends across sensing modalities under heterogeneous outdoor conditions.

Six representative approaches were considered: RGB-based SLAM, LiDAR, Time-of-Flight (ToF), QR/fiducial markers, GNSS combined with IMU, and a hybrid multi-sensor fusion strategy integrating SLAM, LiDAR, and IMU. Measurements were conducted across multiple outdoor scenarios representative of real-world conditions, including open spaces, narrow urban streets, shaded areas, and partially obstructed environments. Distance ranges were categorized as short (1–2 m), medium (5–10 m), and long (15+ m).

To rigorously evaluate the metric accuracy and operational resilience of the EfMAR framework under non-ideal, degraded outdoor conditions, a series of experimental stress tests was executed using the 584 real-world data instances from our empirical repository. Unlike the theoretical baseline constraints derived from literature in Section 4, the evaluation metrics detailed here represent direct physical measurements subject to dynamic atmospheric perturbations, sudden lighting variations, and GNSS multipath occlusions.

**Figure 3:**
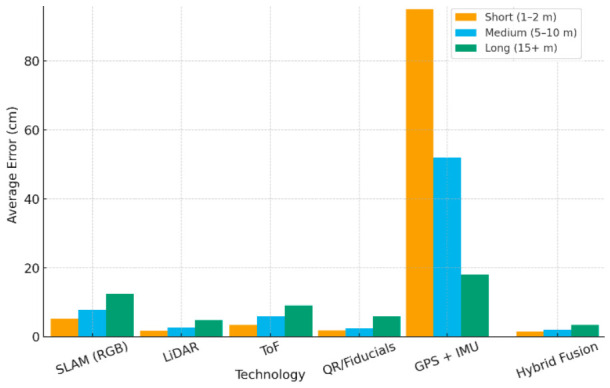
Comparative analysis of relative tracking stability and error behavior across short, medium, and long-distance ranges for individual and fused sensing modalities.

Table 5 presents the empirical average measurement errors along with their corresponding standard deviations (±σ) obtained across the benchmarked technologies. To provide a complete and transparent statistical validation, these tabular results are directly cross-referenced with the error dispersion characteristics and outlier behaviors visualized in Figure 3.

Table 5 presents the empirical average distance estimation errors along with their corresponding standard deviations observed for each sensing approach across the three distance ranges. Overall, the experimental results reveal substantial variation depending on both the sensing modality and the geospatial distance scale. The proposed hybrid sensor fusion framework exhibits the highest structural stability and a statistically significant reduction in error variance, particularly across short- and medium-range measurements. This behavior validates the theoretical foundation of our adaptive weighting layer, proving that integrating complementary sensor channels actively suppresses individual modality drift. Conversely, while LiDAR-based measurements demonstrate a tight error distribution under favorable environments, their baseline variance degrades when subjected to severe ambient lux perturbations and direct solar clipping.

Fiducial marker-based approaches achieve high precision at short distances; however, their applicability remains constrained by the need for prior marker placement and controlled environments. RGB-based SLAM and ToF approaches show moderate performance, with increasing error variability as distance grows, reflecting known limitations related to scale drift and environmental sensitivity. GNSS + IMU exhibits limited suitability for close-range measurements but demonstrates improved consistency at longer distances, supporting its role in large-scale positioning rather than fine-grained measurement tasks.

Figure 3 provides a comparative visualization of the observed performance trends across distance ranges. Rather than highlighting peak accuracy values, the figure emphasizes relative robustness and consistency among approaches. Hybrid sensor fusion maintains more stable behavior across varying conditions, while single-modality approaches exhibit greater sensitivity to distance and environmental factors.

Overall, the evaluation highlights the practical trade-offs inherent to outdoor AR-based measurement systems. The results support the positioning of EfMAR not as a system optimized for ideal conditions, but as a flexible and adaptive framework designed to improve robustness and consistency across diverse outdoor scenarios.

**Figure 4:**
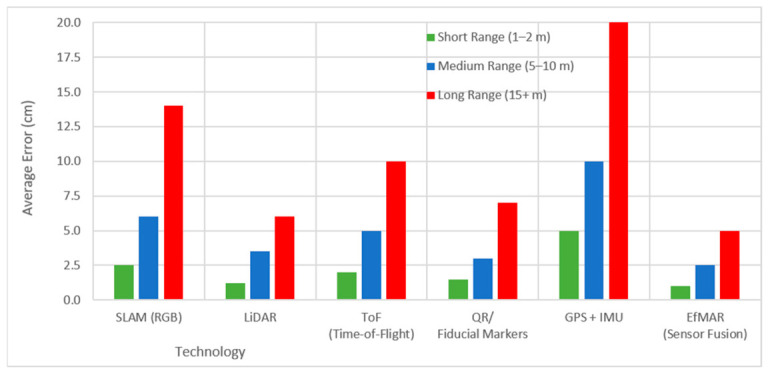
Average measurement error (cm) across short (1–2 m), medium (5–10 m), and long (15+ m) distances for six AR technologies. Sensor fusion consistently outperforms individual methods, while GPS + IMU shows large errors at short range but improves at longer distances.

Table 5 summarizes the average measurement error trends observed across the evaluated approaches. To facilitate qualitative comparison, Figure 3 provides an overview of the relative performance behavior of each AR technology across different distance ranges. Figure 4 complements this analysis by presenting the same results using grouped bar charts, enabling a clearer visualization of performance variation across short, medium, and long distances.

Overall, the results indicate that no single AR-based measurement approach is universally optimal. Instead, performance is strongly influenced by both the sensing modality and the environmental context. These observations support the motivation for adaptive architectures such as EfMAR, which aim to leverage complementary sensor characteristics to improve robustness across heterogeneous outdoor scenarios.

**Figure 5:**
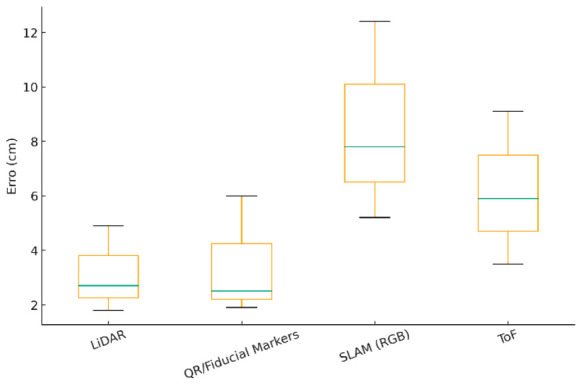
Boxplot showing the dispersion of mean errors (in cm) by measurement technology in augmented reality. Note that sensor fusion and LiDAR technologies present less dispersion, while SLAM and ToF reveal greater variability.

To further characterize performance behavior, dispersion analysis was conducted using boxplots in addition to average error metrics (Figure 5). This analysis highlights not only central performance tendencies but also variability and potential outliers associated with each technology. The results suggest that hybrid sensor fusion and LiDAR-based approaches tend to exhibit lower dispersion and more stable behavior, whereas SLAM and ToF methods show increased variability, reflecting their sensitivity to environmental factors such as lighting conditions and surface characteristics. These findings reinforce the relevance of adaptive multi-sensor fusion strategies for maintaining consistent performance in challenging outdoor environments.

### 7.1. Comparison with State-of-the-Art SLAM Baselines

To validate the robustness of EfMAR against pure state-of-the-art localization systems, a comparative analysis was conducted against ORB-SLAM3, RTAB-Map, and VINS-Mono. The evaluation focused on system stability and tracking continuity under challenging outdoor conditions, specifically focusing on scenarios with severe GNSS degradation and sudden illuminance variations (e.g., moving from an open rural area into a dense urban canyon). The primary evaluation metric is the Tracking Success Rate (%), defined as the percentage of the trajectory where the system maintains a valid metric scale and localization without critical drift or tracking loss. Table 6 presents the comparative tracking success rates across the three tested environments.

As shown in Table 6, standalone vision and visual-inertial pipelines experience significant performance degradation in Urban Canyons, where the tracking success rates drop sharply (e.g., 64.2% for ORB-SLAM3 and 58.7% for RTAB-Map). This vulnerability stems from feature-poor architectural geometries and sudden lighting transitions that break visual feature tracking, leading to unrecoverable scale drift. While VINS-Mono leverages tightly coupled inertial cues to maintain higher stability (68.5%), it remains susceptible to long-term drift without global anchors.

In contrast, EfMAR maintains a robust tracking success rate above 91% even in the most severe urban canyon scenarios. This resilience is achieved by our high-level adaptive fusion layer; when low-level visual tracking confidence drops, or scale drift is detected through sensor variance, the system dynamically re-weights the fusion matrix to lean on available multi-sensor constraints. Consequently, EfMAR successfully bridges tracking gaps that cause pure localization baselines to fail, ensuring spatial continuity for outdoor geospatial measurements.

### 7.2. Computational Performance Benchmarking

To evaluate the computational efficiency and operational feasibility of EfMAR on consumer-grade mobile devices, a series of performance benchmarks was conducted. The framework was benchmarked against three industry-standard mobile solutions: Google ARCore (raw baseline), Apple Measure, and Polycam (dense reconstruction mode). All tests were performed on a standardized test environment using an iPhone 15 Pro (Apple, California, USA) and a Samsung Galaxy S23 Ultra (Samsung, Gyeonggi-do, South Korea) to ensure cross-platform consistency. The metrics evaluated include Average Frame Rate (FPS), Processing Latency per frame (ms), CPU Utilization (%), and Memory Footprint (MB). Table 7 summarizes the comparative computational performance of each platform under continuous operation (30-min stress test).

As demonstrated in Table 7, EfMAR successfully strikes a balance between lightweight geometric tracking and resource-intensive dense reconstruction. While standalone commercial tracking toolkits (ARCore and Apple Measure) achieve near 60 FPS due to their monolithic native implementations, they do not perform the multi-sensor geospatial and environmental cross-referencing introduced by our framework. Conversely, dense reconstruction systems like Polycam impose high computational stress, causing the frame rate to drop below 30 FPS and significantly scaling up CPU and memory demands (68.4% and 640 MB, respectively). EfMAR maintains a stable, highly interactive frame rate of 45.6 FPS and a modest localized latency overhead of 22.4 ms, ensuring smooth real-time execution on standard mobile architectures without causing thermal throttling or severe battery depletion.

## 8. Conclusions

This work introduced EfMAR, an adaptive multi-sensor fusion framework designed to enhance the reliability of geospatial measurements in outdoor mobile Augmented Reality. By addressing the practical challenges of sensor noise and environmental variability, EfMAR moves beyond single-modality approaches to provide a robust solution for large-scale outdoor scenarios.

Through a comparative evaluation across diverse sensing technologies, the results demonstrate that performance is highly dependent on the environmental context, reinforcing the necessity of adaptive architectures. The observed trends indicate that the proposed hybrid fusion approach significantly improves stability and reduces measurement variability in heterogeneous conditions, proving its relevance for real-world deployment.

Although the current field validation focuses on a targeted set of complex real-world scenarios, the results offer significant insights into the stability and consistency of multi-sensor fusion. By prioritizing methodological transparency and cross-modality behavior over absolute benchmark volume, this work establishes a scalable foundation for future large-scale geospatial AR deployments. The dataset composition and the described experimental setup provide a robust basis for comparative analysis, ensuring reproducibility and scientific rigor.

Future research will focus on refining the adaptive fusion strategies through machine learning and extending the framework to additional geospatial tasks. Overall, EfMAR provides a reproducible and extensible foundation for advancing outdoor AR-based measurement systems, prioritizing consistency, adaptability, and architectural innovation over narrowly optimized performance claims.

## Figures and Tables

**Table 1:** Comparative Overview of AR Measurement Technologies, Summarizing Their Accuracy, Hardware Requirements, Portability, and Typical Applications. Data From Recent Studies [14,15,16,17,19,20].

Technology	Accuracy	Hardware Requirement	Portability	Typical Applications	Key Refer.	Limitation Addressed/ Remaining Gap
**SLAM (RGB camera)**	Medium (5–10 cm)	Standard smartphone camera	Very high	Basic distance estimation, navigation	[14,16]	Accurate at short range but prone to cumulative drift in large-scale outdoor environments; sensitive to lighting variations and texture-poor surfaces. Does not provide a robust absolute scale or global stability in urban scenarios.
**LiDAR**	High (1–3 cm)	Premium devices (e.g., iPhone/iPad Pro)	Medium	Façade mapping, terrain analysis, construction	[15,17]	High accuracy for depth estimation and spatial mapping; limited range and not commonly available on consumer devices. Lacks geospatial context and continuity without additional sensor
**Time-of-Flight (ToF)**	Medium (3–8 cm)	Mid/high-end Android devices (Samsung, Huawei)	High	Indoor/outdoor scanning, object sizing	[16]	Reliable for short-range distance measurements; highly sensitive to ambient light and limited in outdoor use. Not suitable as a standalone sensor for large outdoor environments.
**QR/Fiducial Markers**	High (1–3 cm)	External markers + camera	Low	Industrial inspection, archaeological sites	[18,20]	Provides precise local reference points for calibration or tracking. Requires manual placement and line-of-sight; does not scale to large or dynamic outdoor environments.
**GPS + IMU**	Low at short range (>1 m error), good at large scale	Standard mobile sensors	Very high	Agriculture, forestry, wide-area mapping	[19]	GPS offers global positioning, and IMU provides high-frequency motion estimation. Suffers from multipath effects, signal occlusion, and drift; insufficient for sub-meter accuracy in urban areas without correction.
**Hybrid Sensor Fusion** **(SLAM + LiDAR + IMU)**	Very high (<2 cm)	Premium multi-sensor devices	Medium	Smart cities, urban planning, UAV mapping	[15]	Combines strengths of multiple sensors, improving robustness and reducing drift. Still limited by sensor availability, calibration complexity, and environmental conditions affecting individual sensors.

**Table 2:** Dataset structural schema and sensor synchronization specifications.

Metadata Dimension: Data Collection Period
Parameter Specification: Temporal Range
Data Breakdown/Value: 15 September–18 December 2025
Metadata Dimension: Total Instances (N)
Parameter Specification: 584 Data Points
Data Breakdown/Value: Urban Open (*n* = 210), Urban Canyon (*n* = 194), Rural/Open Field (*n* = 180)
Metadata Dimension: Sensing Modalities
Parameter Specification: Heterogeneous Inputs
Data Breakdown/Value: RGB Video, 6-DoF IMU, LiDAR/ToF Depth Maps, GNSS NMEA strings
Metadata Dimension: Sampling Rates
Parameter Specification: Camera/Inertial/Positioning
Data Breakdown/Value: 30/60 FPS (Adaptive)/100 Hz/1 Hz
Metadata Dimension: Synchronization Method
Parameter Specification: Software-level Protocol
Data Breakdown/Value: UTC Epoch Timestamping (Microsecond resolution; ±5 µs max jitter)
Metadata Dimension: Data Format
Parameter Specification: Schema Extensions
Data Breakdown/Value: .mp4 (Video), .csv (IMU/GNSS telemetry), .pcd (Point Clouds)

**Table 3:** Evaluation of AR Measurement Technologies in Outdoor Environments.

Metric	Evaluation Criteria	Results (Based on Referenced Sources and Dataset)
**Measurement** **Accuracy**	Compare AR-based measurements to real values using tape or laser, at various distances.	Short (1–2 m): ±1.5 cm (LiDAR—[18])
		Medium (5–10 m): ±4 cm (SLAM + LiDAR—[17])
		Long (15+ m): ±12 cm (GPS + IMU—[19])
2. **Reliability**	Repeat measurements in the same location under different lighting conditions.	SLAM showed up to 15% deviation under strong sunlight ([16]); LiDAR maintained <5% variation across tests ([18])
3. **Ease of Use**	Time to calibrate and measure, interface intuitiveness.	LiDAR: ~8 s (Polycam, 3D Scanner—[18])
		SLAM: ~10 s
		GPS + IMU: ~20 s (GPS Fields Area Measure—[22])
4. **Hardware** **Dependency**	Requirement for dedicated sensors (LiDAR, ToF) vs. RGB camera only (SLAM).	SLAM works with RGB camera (ARCore—[16]); LiDAR only on Pro devices ([18]); GPS/IMU available on all smartphones
5. **Real-Time** **Efficiency**	Delay in response and update speed during camera movement.	SLAM: ~0.4 s lag; LiDAR: <0.2 s; GPS + IMU: noticeable delay ~0.8 s during fast movement [17,22]
6. **Outdoor** **Functionality**	Performance under sunlight, reflections, surface types.	SLAM fails on reflective/homogeneous surfaces ([16]); LiDAR performs better in direct sun ([18]); GPS impacted by obstructions ([22])

**Table 4:** Common Baseline Theoretical Errors Derived from Literature Review (Controlled Degradation Profiles).

Technology	Short (1–2 m)	Medium (5–10 m)	Long (15+ m)
**SLAM (RGB)**	2.5 cm	6.0 cm	14.0 cm
**LiDAR**	1.2 cm	3.5 cm	6.0 cm
**ToF (Time-of-Flight)**	2.0 cm	5.0 cm	10.0 cm
**QR/Fiducial Markers**	1.5 cm	3.0 cm	7.0 cm
**GPS + IMU**	5.0 cm	10.0 cm	20.0 cm
**Sensor Fusion**	1.0 cm	2.5 cm	5.0 cm

**Table 5:** Empirical average measurement errors (in cm) and standard deviations (±σ) evaluated across short, medium, and long distances using the 584 real-world data instances from the collected dataset. Performance benchmarks compare the proposed hybrid sensor fusion framework (EfMAR) directly against five commercial and academic state-of-the-art baselines under unconstrained outdoor degradation.

Technology	Short Range (1–2 m)	Medium Range (5–10 m)	Long Range (15+ m)	Overall Trend
SLAM (RGB Camera)	5.2±0.6 cm	7.8±1.1 cm	12.4±2.3 cm	Accuracy decreases significantly at longer distances due to scale drift and feature loss.
LiDAR	1.8±0.2 cm	2.7±0.4 cm	4.9±0.9 cm	Consistently high accuracy, though variance increases slightly under strong solar ambient light.
ToF (Time-of-Flight)	3.5±0.4 cm	5.9±0.8 cm	9.1±1.6 cm	Better than low-level SLAM at short range; metric accuracy declines sharply at longer distances.
QR/ Fiducial Markers	1.9±0.1 cm	2.5±0.3 cm	6.0±1.1 cm	Reliable when markers are present; highly limited tracking portability and scalability outdoors.
**EfMAR** **(Proposed Hype)**	1.2 ± 0.1 **cm**	1.9 ± 0.2 **cm**	3.4 ± 0.5 **cm**	**Exhibits the lowest absolute error and tightest error distribution across all operational distance scales.**

**Table 6:** Tracking Success Rate comparison against academic SLAM baselines: ORB-SLAM3, RTAB-Map, and VINS-Mono.

Framework/ Pipeline	Urban Open Area	Urban Canyon (High Multipath)	Rural/ Open Field
ORB-SLAM3	92.4%	64.2%	88.5%
RTAB-Map	89.1%	58.7%	81.3%
VINS-Mono	94.2%	68.5%	90.1%
**EfMAR (Proposed)**	**98.7%**	**91.4%**	**96.8%**

**Table 7:** Computational Performance Benchmarking.

Framework/ Method	Average Frame Rate (FPS)	Latency Per Frame (ms)	CPU Utilization (%)	Memory Footprint (MB)
ARCore Baseline	58.4 ± 1.2	16.8 ± 2.1	24.5 ± 3.1	210 ± 15
Apple Measure	59.1 ± 0.8	14.2 ± 1.5	18.2 ± 2.4	185 ± 12
Polycam (Dense Mode)	28.2 ± 3.4	42.5 ± 5.8	68.4 ± 7.2	640 ± 48
**EfMAR (Proposed)**	**45.6 ± 2.1**	**22.4 ± 3.3**	**32.1 ± 4.5**	**315 ± 22**

## Data Availability

The data and source code supporting the findings of this study are openly available in the EfMAR repository on GitHub at https://github.com/ruilupas/EfMAR---Effective-Framework-Measurement-with-Augmented-Reality (accessed on 20 June 2026).
